# Surgical treatment of primary pulmonary artery sarcoma

**DOI:** 10.1007/s11748-020-01476-2

**Published:** 2020-09-12

**Authors:** Yongxin Han, Yanan Zhen, Xiaopeng Liu, Xia Zheng, Jianbin Zhang, Zhenguo Zhai, Jun Duan, Yajun Zhang, Peng Liu

**Affiliations:** 1 Peking University China-Japan Friendship School of Clinical Medicine, Beijing, 100029 China; 2 Department of Cardiovascular Surgery, China-Japan Friendship Hospital, Cherry Park East Street, Chaoyang District, Beijing, 100029 China; 3 Respiratory Department, China-Japan Friendship Hospital, Beijing, China; 4 Surgical Intensive Care Unit, China-Japan Friendship Hospital, Beijing, China; 5 Surgical Anesthesia Department, China-Japan Friendship Hospital, Beijing, China

**Keywords:** Pulmonary artery sarcoma, Pulmonary thromboendarterectomy, Postoperative chemotherapy

## Abstract

**Objective:**

Primary pulmonary artery sarcoma (PAS) is a rare tumor that originates from the intimal layer of the pulmonary artery or pulmonary valve and has a poor prognosis. The standard treatment for this devastating disease remains unclear. This study aimed to summarize the current standard treatments for PAS.

**Methods:**

From September 2015 to January 2020, six patients were diagnosed with PAS and underwent pulmonary endarterectomy (PEA) at our department. Their medical records were retrospectively reviewed to analyze the clinical characteristics, histopathological features, and postoperative outcomes. Fourteen articles, each reporting at least 6 cases, identified 201 patients diagnosed with PAS, and 158 patients had detailed treatments and follow-up data.

**Results:**

All of the patients who successfully underwent PEA were alive at follow-up, with a mean survival duration of 11.6 months (7–28 months), and one patient developed recurrence in the right upper lobe lung. Two patients received postoperative chemotherapy. In one patient, the tumor invaded the pulmonary valve.

**Conclusions:**

PAS resection combined with PEA via the aid of cardiopulmonary bypass and deep hypothermic circulatory arrest could achieve maximal tumor resection in patients without metastatic lesions. An individualized surgery strategy relies on a precise preoperative imaging examination. Moreover, postoperative adjuvant therapy could yield improved survival outcomes.

## Introduction

Pulmonary hypertension (PH) is defined as a mean pulmonary artery pressure (mPAP) ≥ 20 mmHg at rest as assessed by right heart catheterization [1], and pulmonary artery sarcoma (PAS) along with chronic thromboembolic pulmonary hypertension (CTEPH) is categorized as Group 4 PH because of the similar hemodynamic index [2] and a pulmonary artery wedge pressure (PAWP) ≤ 15 mmHg. Transthoracic and transesophageal echocardiography (TTE and TEE), computed tomography pulmonary angiography (CTPA), cardiac magnetic resonance imaging (MRI), endobronchial ultrasound transbronchial needle aspiration (EBUS-TBNA) and fluorodeoxyglucose positron emission tomography/CT (FDG-PET/CT) are necessary for the diagnosis of PAS [3–6]. The prognosis of PAS is poor, and the median survival time without surgical resection was reported to be 1.5 months [3], while surgical resection of the tumor could lengthen the survival time to 8–36 months [7]. The main PAS treatment strategy is still unclear because of the limited number of reported cases in the literature.

We report our experience and review the published cases to increase the understanding of PAS and to improve treatment modalities.

## Methods

From September 2015 to June 2020, 63 patients were diagnosed with PH and transferred to our department for surgical treatment, including 57 with CTEPH and 6 with PAS. We retrospectively reviewed their clinical records to evaluate the patient characteristics, perioperative findings, and postoperative outcomes. There were 14 articles, which reported at least 6 each, that identified 201 patients diagnosed with PAS, and 158 patients had detailed treatment and follow-up data [7–20].

## Results

### Patient characteristics

Six patients (5 women and one man; median age 52 years old, ranging from 33 to 52) were identified as having PAS via postoperative pathologic examination between September 2015 and June 2020. The patient characteristics are summarized in Table 1.

**Table 1:** Patient characteristics (*n* = 6)

Variables	Number of patients
Gender	
Female	5(6)
Median age (range)	52 (33–52)
Clinical manifestations	
Dyspnea	6
NYHA class	
I, II	2
III, IV	4
Chest pain	2
Syncope	1
hemoptysis	1
Constitutional symptoms	
Fever	4
Fatigue	5
Weight loss	3
Preoperative workup	
TTE	6
CTPA	6
MRI	5
Pulmonary angiography	5
PET/CT	4
Biopsy	1

*TTE* transthoracic echocardiography, *CTPA* computed tomography pulmonary angiography, *MRI* magnetic resonance imaging, *PET/CT* positron emission tomography computed tomography

The preoperative workups included TTE (*n* = 6), CTPA (*n* = 6), MRI (5), pulmonary angiography (*n* = 4) and PET/CT (*n* = 4). Biopsy was performed in 2 patients via EBUS-TBNA (no. 2) and ultrasonography-guided percutaneous biopsy of the lung nodules (no. 5). Four patients were misdiagnosed with CTEPH (*n* = 3) and pulmonary embolism (*n* = 1), with a mean duration of misdiagnosis of 10.5 months.

### Radiologic investments

All the patients received CTPA, which showed filling defects within the pulmonary artery (PA) with soft-tissue characteristics. Enhanced MRI was performed in five patients. The manifestations were as follows: (1) pulmonary trunk (PT) and/or right and/or left pulmonary artery (RPA or LPA) filling defects showing a high T2 signal (*n* = 5); (2) a mass showing a high signal on diffusion-weighted imaging (DWI) (*n* = 5); (3) heterogeneous enhancement of the mass (*n* = 3); (4) no hypertrophy of the right ventricular mass (*n* = 5); and (5) pulmonary valve (PV) involvement (*n* = 1).

EBUS-TBNA was attempted in patient no. 2, and a definite preoperative diagnosis of PAS followed the postoperative pathological diagnosis. No biopsy of the PA mass was made via right ventricle catheterization during pulmonary angiography.

Four patients underwent PET/CT examinations. All PA masses typically had high standardized uptake values (SUVs).

### Surgical techniques

Median sternotomy with the aid of cardiopulmonary bypass (CPB) was routinely performed. Intraoperative examination confirmed the tumor location in the main pulmonary artery (MPA) (*n* = 5), bilateral pulmonary artery (BPA) (*n* = 3), LPA (*n* = 2), RPA (*n* = 2) and PV (*n* = 1). Appositional thrombosis covering the tumor and segmental artery thrombosis were found simultaneously in patient no. 1, 2, and 3, who had a longer duration of misdiagnosis than patient no. 5 who only had an isolated tumor. Pulmonary endarterectomy (PEA) was managed via deep hypothermic circulatory arrest. The detailed deep hypothermic circulatory arrest (DHCA) strategy is shown in Table 2. Patient no. 4 had PV involvement with a continuously lumpy intima from the tumor base to the PV. Therefore, PV resection combined with PEA was performed. Then, one single leaflet was reconstructed from the pericardium with a 5-0 polypropylene suture. All arteriotomies were closed with a continuous 6-0 polypropylene suture.

**Table 2:** DHCA strategy

Patient	Cardiopulmonary bypass time (min)	Cross-clamping time (min)	DHCA					
			Temperature (℃)			Circulatory arrest		Circulatory arrest time (min)
			Tympanum	Anus	Urocyst	Frequency	Time (min)	
1	321	136	18.6	20.5	20.5	3	20 + 20 + 4′40	44′40
2	235	135	15.7	18.4	18.6	2	15′30 + 8′40	24′10
3	403	196	16.2	19.8	19.3	4	18′50 + 20 + 6′56 + 8′04	53′51
4	330	173	18	19.8	19.8	4	20 + 20′10 + 20 + 20′10	80′20
5	222	96	17	20.5	19.6	2	13′36 + 4′20	17′56
6	346	150	18	19.8	19.8	4	20 + 10′29 + 20 + 7′20	57′49

*DHCA* deep hypothermic circulatory arrest

**Fig. 1:**
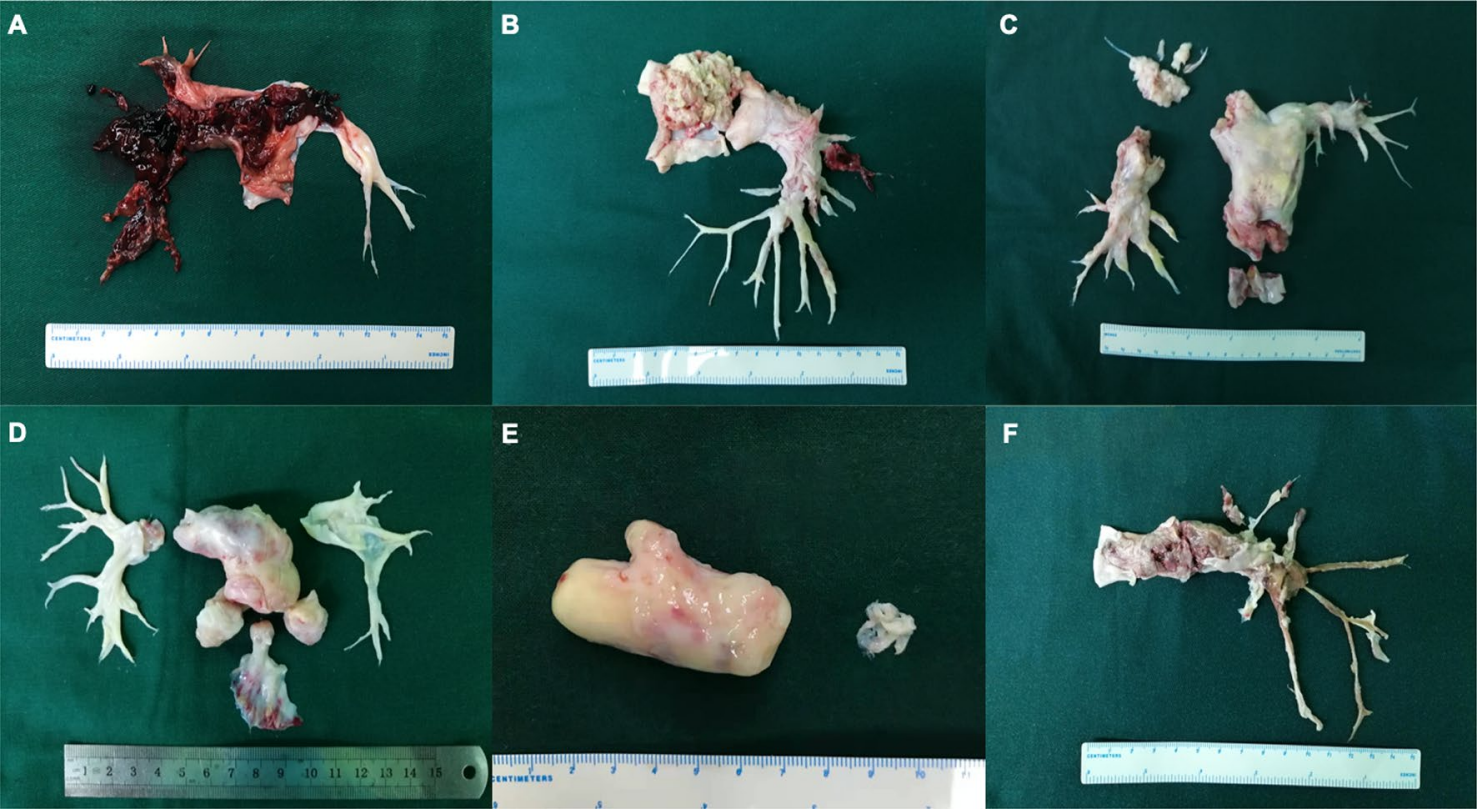
Surgical specimens. Surgical specimens from patient no. 1–6 are showed in **a**–**f** in order

Fast frozen sections of the pulmonary mass were routinely obtained to confirm complete resection. The distal resection margin from the RPA was detected to have tumor cells in patient no. 2. The endarterectomy plan could not be re-established because the intima retracted distally once resection of the suitable intima was completed. Right pneumonectomy was not considered. All of the surgical specimens are shown in Fig. 1.

### Pathological results

Paraffin-embedded tissue specimens were available in all 6 cases. Immunohistochemical staining for cytokeratin (CK), S-100 protein, desmin, and smooth muscle actin was performed. Three specimens were diagnosed as pleomorphic fascicular sarcoma with focal myogenic differentiation, two specimens were classified as sarcoma with osteosarcomatous or chondrosarcomatous differentiation, and one patient was diagnosed with leiomyosarcoma.

### Clinical results

There were no perioperative deaths. Four patients received postoperative chemotherapy with a regimen of Adriamycin (A) plus ifosfamide (I), and patient no. 2 developed a recurrence in the right lung 4 months after the operation and received no adjuvant therapy in their local referring hospital. The specific postoperative outcomes are summarized in Table 3.

**Table 3:** Treatments and postoperative outcomes

Patient	Gender/age	Misdiagnosis/month	Surgicacl type	Tumor location	PAP(mmHg)		Fast frozen section	Histologic type	Chemotherapy	Recurrence	Survival (month)
					Pre-operation	Post-operation					
1	F/39	CTEPH/7	PEA	MPA + BPA	55	30	−	Pleomorphic fascicular sarcoma with focal myogenic differentiation	A + I	None	28 (alive)
2	F/52	CTEPH/21	PEA	MPA + LPA	44	37	+	Pleomorphic fascicular sarcoma with focal myogenic differentiation	None	Right upper lobe lung	20(alive)
3	M/54	PE/4	PEA	MPA + BPA	40	35	−	Leimyocarcoma	None	None	15 (alive)
4	F/49	CTEPH/9	PV resection + PEA	MPA + BPA + PV	60	39	−	Sarcomsa with osterosarcomatous differentiation	A + I	None	11(alive)
5	F/54	−	PEA	MPA + RPA	55	38	−	Fascicular sarcoma	A + I	None	11 (alive)
6	F/33	−	PEA	MPA + LPA	40	33	−	Sarcoma with chondrosarcomatoue differentiatiion	A + I	None	7 (alive)

*F* female, *M* male, *PAS* pulmonary artery sarcoma, *PEA* pulmonary endarterectomy, *MAP* main pulmonary artery, *BPA* bilateral pulmonary artery, *RPA* right pulmonary artery, *LPA* left pulmonary artery, *A* adriamycin, *I* ifosfamide, *PVR* pulmonary valve reconstruction, *SPAP* systolic pulmonary artery pressure

## Discussion

In 1923, Mandelstamm reported the first PAS via autopsy, and since then, approximately 400 cases have been described, mostly as case reports [8]. The incidence of PAS was reported as 0.001–0.03% based on autopsy [21], but this incidence might be underestimated because of the insidious onset of PAS and misdiagnoses as any cause of obstructive pulmonary hypertension, such as pulmonary embolism (PE) and CTEPH, primary lung cancer and extra thoracic tumor emboli extending into the PA [13].

According to the reported cases in the literature, PAS is often misdiagnosed as CTEPH owing to their common PA occlusion symptoms, including dyspnea, chest pain, hemoptysis, and syncope. Pu et al. summarized the constitutional symptoms of PAS, including fever, fatigue, and weight loss [9].

### Radiologic investigations

The CTPA characteristics of PAS include hyperdense lesions with nonhomogeneous attenuation from hemorrhage, beaded peripheral PA, contiguously soft-tissue-filled PA, an entirely occupied lumen, vascular distention from tumor growth, distal oligemia, and extravascular spread; these findings could help to locate the tumor preoperatively [15]. MRI (especially enhanced MRI) and PET/CT could help to differentiate PAS from pulmonary thromboembolic disease (PTD).

MRI can detect PAS soft tissue within the lumen of the PA and help reveal involvement between the tumor and PV because of its excellent spatial and tissue resolution [22, 23]. Moreover, enhanced MRI could facilitate the differentiation of PAS from a thrombosis via the specific imaging characteristics of PAS, including strong gadolinium enhancement, hyperintensity, especially on fat-suppressed T-weighted imaging and DWI, and a thickened PA intima adjacent to the tumors with delayed enhancement [24]. Enhanced MRI could not distinguish the structures of the intima, media, and adventitia in the traditional sense in our center. What can be distinguished is whether the tumor breaks through the vascular wall and infiltrates the tissues or organs around the blood vessels, because there are apparent signal differences between the vascular wall and the surrounding tissues. Nevertheless, many patients with PAS have difficulty holding their breath long enough to allow for MRI scanning, limiting the use of MRI for the diagnosis of PAS [9]. Furthermore, for identification with PET/CT, the SUV measurements of PAS are higher than those of thrombotic lesions because of the hypermetabolic nature of tumor cells [25, 26].

### Surgical treatment of PAS

Surgical resection can alleviate clinical symptoms, offer adequate palliation and increase survival [20, 27]. The mean survival duration without surgery was 1.5 months after diagnosis, and surgical intervention could lengthen this survival time to 10 months, according to Kruger [27].

#### Surgery strategy

Owing to the rarity of PAS and limited small-scale case reports represented in the literature, there is still no standard surgical procedure. Blackmon et al. [15] proposed a staging system for primary PAS in terms of preoperative imaging evidence: stage I: tumor limited to the main pulmonary artery; stage II: tumor involving one lung plus a main PA; stage III: bilateral lung involvement; and stage IV: extra thoracic spread. They also recommended criteria for surgery, including an adequate cardiopulmonary reserve, lack of disease in the chest, resectable disease, and an adequate lung function reserve if pneumonectomy is considered. Patients not considered candidates for curative resection may also benefit from palliative resection.

#### Pneumonectomy

Before the twenty-first century, pneumonectomy with or without PA replacement was the mainstay surgical procedure [17, 20, 27, 28]. The overall unilateral PAS rate is only 30% (44/145) among the reported cases [7, 9–12, 14–18]. Grazioli et al. concluded that pneumonectomy might be a possible radical resection technique for the base of unilateral disease, achieving a median survival 26.8 months longer than the 6.6 months in the PTE group [12]. However, given the high probability of cancer dissemination secondary to vascular disease and the high possibility of bilateral tumor cell seeding with the surgical procedure, unilateral pneumonectomy might not be adequate [11].

##### PEA

With increasing understanding of the pathophysiological process of PAS, which includes an origin from the pulmonary artery intima and invasion in a proximal, distal, and centrifugal manner [13], tumor resection with bilateral PEA might be an effective procedure. Mussot et al. retrospectively reviewed 31 patients with operable PAS. They found that the 20 patients receiving PEA alone seemed to have a more prolonged survival duration than the 11 patients who underwent pneumonectomy [13]. Gan et al. [14] concluded that only PAS resection without distal segmental pulmonary embolectomy might leave the superimposed thrombosis and metastasized PAS emboli, which lead to progressive obstruction in the distal segmental PA [29]. In their study, the median survival time of the five patients without distal embolectomy was 10 months; in contrast, the four patients who underwent distal embolectomy achieved 30–43 months of survival at follow-up. Therefore, PEA might be an option to achieve complete tumor resection.

#### PEA combined with pneumonectomy

The use of PEA combined with pneumonectomy is still controversial. In Mussot’s study, two patients suffered from adult respiratory distress syndrome following pneumonectomy, and two patients among the five receiving PEA combined with pneumonectomy underwent reoperation owing to postoperative hemorrhage. We did not perform PEA synchronically with right pneumonectomy in patient no. 2 in consideration of the insufficient reserved respiratory function; this patient was a potential candidate for aggressive postoperative adjuvant therapy.

##### DHCA

The aim of CPB and DHCA is to stop significant back-bleeding from systemic neovascularization in the lung from bronchial arteries [13] after identifying the correct plane for endarterectomy. However, DHCA could damage our normal physiological process. Yin and colleagues [8] performed distal embolectomy routinely via the aid of CPB, but DHCA was not performed in their series. In Deng’s study [7], 13 patients underwent PTE; among these patients, DHCA was applied in 3 patients, deep hypothermic low flow was used in 6 patients, and the other four patients received mild or moderate CPB. In our department, DHCA was applied in every PEA patient, including those with CTEPH and PAS, and the DHCA strategy is shown in Table 3. The clearer the operative field is, the more sufficiently we could achieve true endarterectomy during the PEA procedure to achieve maximum tumor resection. More robust studies are needed to confirm whether deep hypothermic low flow or mild or moderate CPB without circulatory arrest leads to a better prognosis than DHCA.

#### Pulmonary valve involvement

**Fig. 2:**
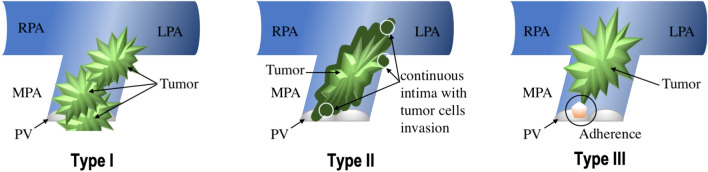
Illustration of three types of PV involvement. Three types of PV involvement situations. Type I: tumor locating in both RVOT or PV and PAs; Type II: tumor locating in PAs extending to the PV in a retrograde manner within the intima from tumor basal to the annulus of PV; Type III: tumor locating in the PAs adhering the PV margin without a continuous intima invasion

The PV was involved in approximately 30% of PAS cases reported in the literature [13, 28]. Blackmon thinks that if PV excision is necessary for R0 resection, then a homograft can be used for PV reconstruction [15]. Gan et al. peeled the tumor carefully from the PV and PA without PV reconstruction [14]. Furthermore, Deng et al. performed 3 PEA combined with PV replacement (PVR) for six patients with PV involvement [7], and the patients who underwent PVR achieved a longer survival time (29.3 months vs. 7 months). Patient no. 4 had PV involvement with a continuous lumpy intima from the tumor base to the PV. Therefore, PV and PAS resection combined with bilateral PEA was performed. Then, one single leaflet was reconstructed from the pericardium with a 5–0 polypropylene suture. According to the reported cases in the literature and one of our cases with PV involvement, we summarized three types of PV involvement situations during surgery, as illuminated in Fig. 2. Moreover, we proposed suggestions to handle the different situations by reviewing the literature: type I: ROVT incision, PV resection, and right ventricular outflow tract (RVOT) or PV reconstruction with PEA; type II: PA trunk incision, PV resection, and reconstruction with tumor resection and PEA; and type III: PA incision, tumor resection, and PEA. The surgical strategy should be individualized and combined with the surgeons' own operative experiences. Different PV reconstructions in the field of PAS are worth further clinical validation.

### Adjuvant therapy

Neoadjuvant therapy could theoretically permit tumor shrinkage, enhance resectability, and potentially neutralize micrometastatic disease [15]. However, few patients can tolerate the side effects of chemo/radiotherapy owing to their impaired hemodynamics. Postoperative tumor recurrence was inevitable in the literature and in our center. The reported recurrence sites show that local sites (14/89) are the most common location, followed by the lung (11/89), brain (4/89), and liver (3/89) [7, 8, 13–15, 17, 19]. The reported cases proclaimed a benefit from postoperative adjuvant therapy (chemo/radiotherapy) by possibly neutralizing circulatory tumor metastasis to reduce the recurrence rate. We analyzed the presented data on survival time, comparing patients who received adjuvant therapy with those who did not receive adjuvant therapy. There were 45 patients in the postoperative adjuvant therapy cohort and 16 in the other cohort, and the mean survival times were 23 ± 18 months and 14 ± 13 months, respectively, with no significant difference (*P* = 0.06) [7, 14, 15, 17–20] (the reported data and analyzed results are shown in Table 4). The trend towards longer a survival time with postoperative adjuvant therapy is evident but more clinical data should be collected more reliable evidence [30]. Nevertheless, there were not enough patients and only one type of chemotherapy regimen, so we could not determine any benefit [10, 11, 15].

**Table 4:** Summarized outcomes between the adjuvant therapy group and the other

Study	Neo/adjuvant		None		*P*-value
	*n*	total survival time (month)	*n*	Total survival time (month)	
Anderson et al. [20]	5	51	1	5	
Mayer et al. [19]	6	162	1	9	
Kim et al. [4]	5	104	4	50	
Blackmon et al. [15]	8	284	0	-	
Gan et al. [14]	7	166	2	46	
Deng et al. [7]	8	99	3	58	
Huo et al. [18]	6	176	5	50	
Average	45	23 ± 18	16	14 ± 13	0.06

## Conclusions

PEA operation via the aid of CPB and DHCA could achieve maximal tumor resection in patients without metastatic lesions. An individualized surgery strategy relies on a precise preoperative imaging examination. Moreover, postoperative adjuvant therapy could yield improved survival outcomes.

### Limitations

There were only six patients in the study. Consistent with the literature, the incidence rate of PAS was low; the number of PAS patients within a single surgical center is limited, which leads to a low level of evidence. Moreover, in our study, we missed the preoperative evaluation of the tumor invasion depth because of the limitation of the imaging technique. Therefore, standardized case reports are conducive to furthering our understanding of PAS and facilitating more comprehensive research.
